# The efficacy of right ventricular pacing for symptomatic left mid‐ventricular obstruction

**DOI:** 10.1002/joa3.12951

**Published:** 2023-11-07

**Authors:** Sou Takenaka, Akihiko Ueno, Daisuke Iida, Masayoshi Sakakibara

**Affiliations:** ^1^ Department of Cardiology IMS Katsushika Heart Center Tokyo Japan

**Keywords:** hypertrophic obstructive cardiomyopathy, implantable cardioverter defibrillator, left mid‐ventricular obstruction, pacing

## Abstract

The serial changes in intraventricular pressure gradient in the left ventricle and NYHA functional classification in each case. Both the left intraventricular pressure gradient and symptoms improved after right ventricular pacing. In one case, the left intraventricular pressure gradient disappeared immediately after right ventricular pacing, while in the others it disappeared during the chronic phase, more than a year later.

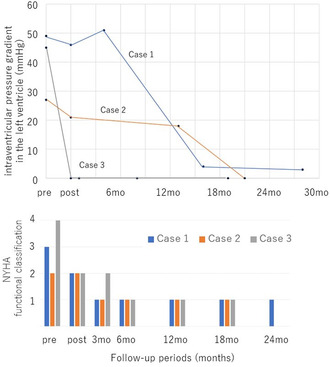

The effect of right ventricular pacing therapy for hypertrophic obstructive cardiomyopathy (HOCM) with left ventricular outflow tract (LVOT) obstruction is well documented, particularly in reducing intraventricular pressure gradients in the left ventricle during the chronic phase.^1^ However, reports on pacing therapy for left mid‐ventricular obstruction (LMVO) are limited.^2^, ^3^ Here, we describe three cases of patients with LMVO who underwent right ventricular pacing therapy.

The first case (Figure 1) is a 75‐year‐old man who suffered from faintness and palpitations during exercise, and presented to our hospital. An electrocardiogram revealed repetitive nonsustained ventricular tachycardia (Figure 1B), and an ultrasonic echocardiography (UCG) (Figure 1C) revealed LMVO (16 mm thickness) with an intraventricular pressure gradient of approximately 49 mmHg in the left ventricle. A dual chamber‐implantable cardioverter defibrillator (ICD) was implanted (HCM risk‐SCD calculator: 4.41%/5 years), and the atrioventricular (AV) delay was shortened to right ventricular pacing (Figure 1D). Symptoms resolved after pacing therapy, and echocardiography 2 years later revealed a pressure gradient resolution (3 mmHg) and myocardial thickening improvement to 13 mm. Three years have elapsed since ICD implantation without ICD therapy.

**FIGURE 1:**
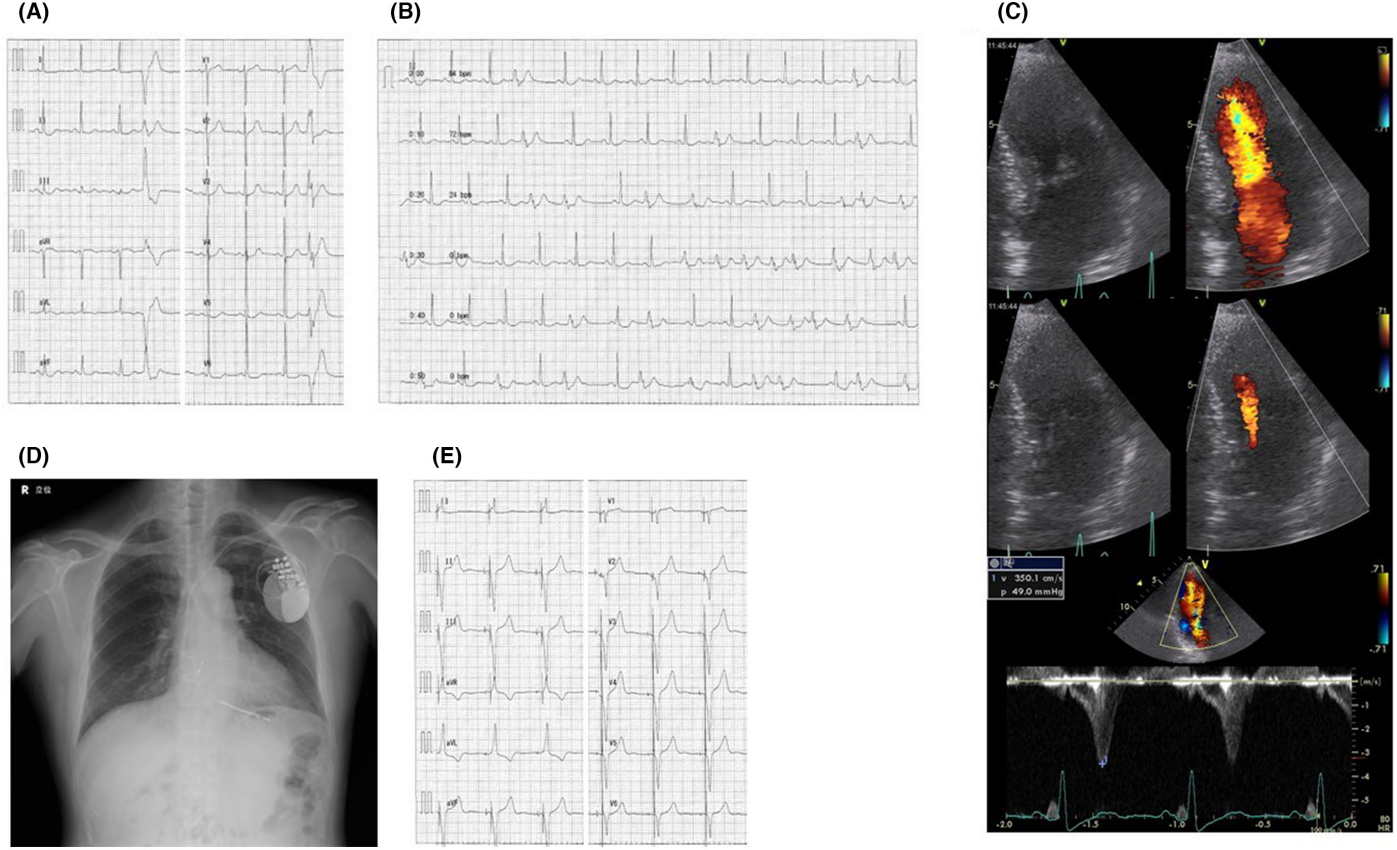
(A) The 12‐lead electrocardiogram (ECG) during sinus rhythm. (B) ECG of nonsustained ventricular tachycardia. (C) Ultrasonic echocardiography (UCG) revealing left mid‐ventricular obstruction with a 16 mm maximum left ventricular wall thickness and a 49 mmHg intraventricular pressure gradient. (D) Chest x‐ray after ICD implantation. The right ventricular lead was placed in the right ventricular apex. (E) ECG during RV pacing.

The second case (Figure 2) is a 73‐year‐old man who had shortness of breath upon exertion and fainted twice before during exercise. A UCG revealed LMVO (17 mm thickness) with an intraventricular pressure gradient of 27 mmHg (Figure 2B). The left ventricular pressure gradient decreased to 15 mmHg 1 month after ICD implantation and pacing therapy (HCM risk‐SCD calculator: 4.76%/5 years) (Figure 2C), and disappeared after 1.5 years. He has not experienced syncope since then. Two years have elapsed since ICD implantation without ICD therapy.

**FIGURE 2:**
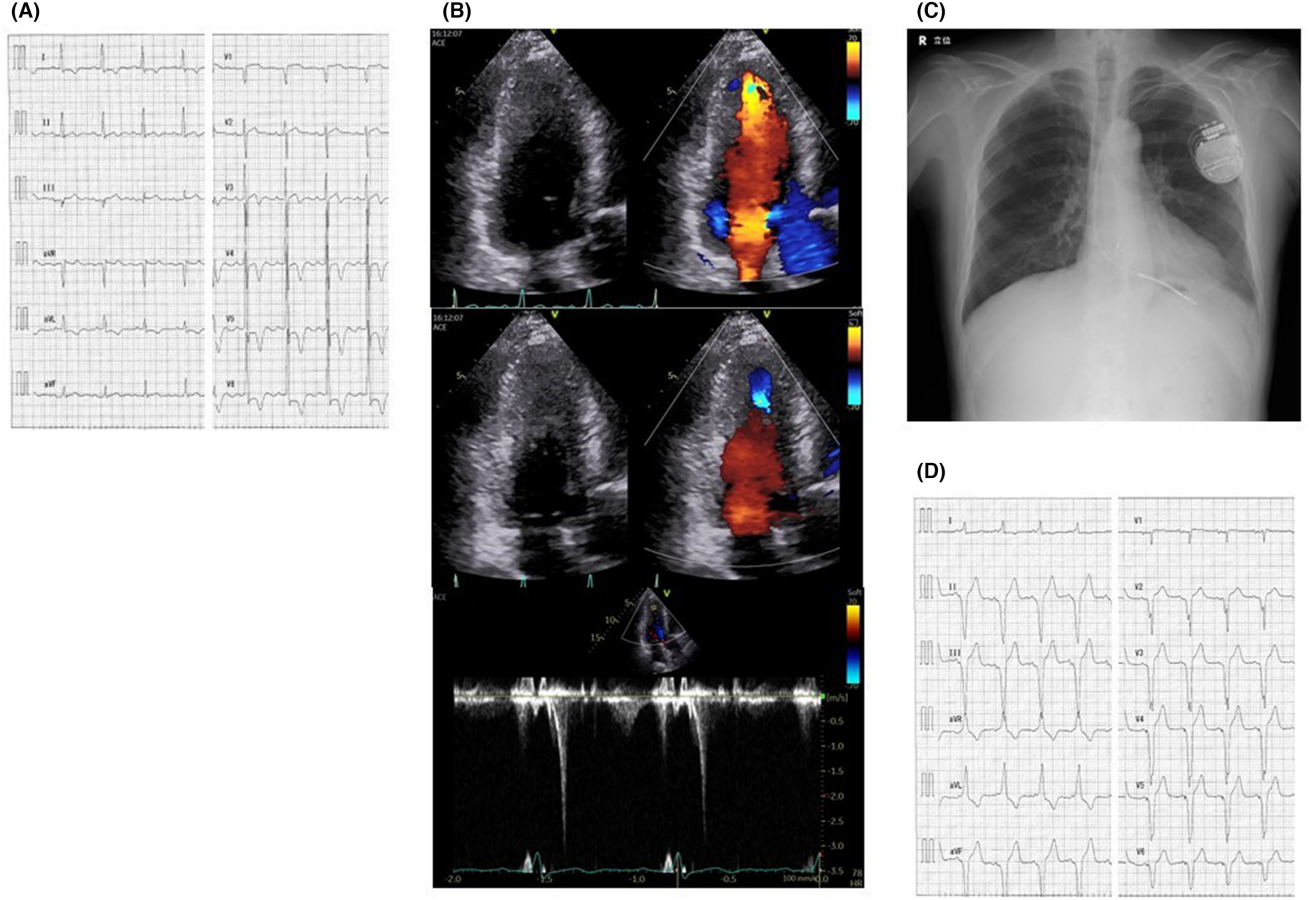
(A) The 12‐lead electrocardiogram (ECG) during sinus rhythm. (B) Ultrasonic echocardiography (UCG) revealing left mid‐ventricular obstruction with a 17 mm maximum left ventricular wall thickness and a 27 mmHg intraventricular pressure gradient. (C) Chest x‐ray after ICD implantation. The right ventricular lead was placed in the right ventricular apex. (D) ECG during RV pacing.

The third case (Figure 3) is a 74‐year‐old man who was referred for shortness of breath upon exertion. He had persistent atrial fibrillation, and a Holter electrocardiogram revealed nonsustained ventricular tachycardia (Figure 3B). A UCG revealed LMVO (24 mm thickness) with an intraventricular pressure gradient of approximately 45 mmHg (Figure 3C). The apex of the left ventricle was aneurysmal and thinning. ICD implantation was performed (HCM risk‐SCD calculator: 4.79%/5 years) and an AV block was created to prevent ICD malfunction and right ventricular pacing (Figure 3D). The intraventricular pressure gradient disappeared and shortness of breath on exertion improved postoperatively. One and a half years have elapsed since ICD implantation without ICD therapy.

**FIGURE 3:**
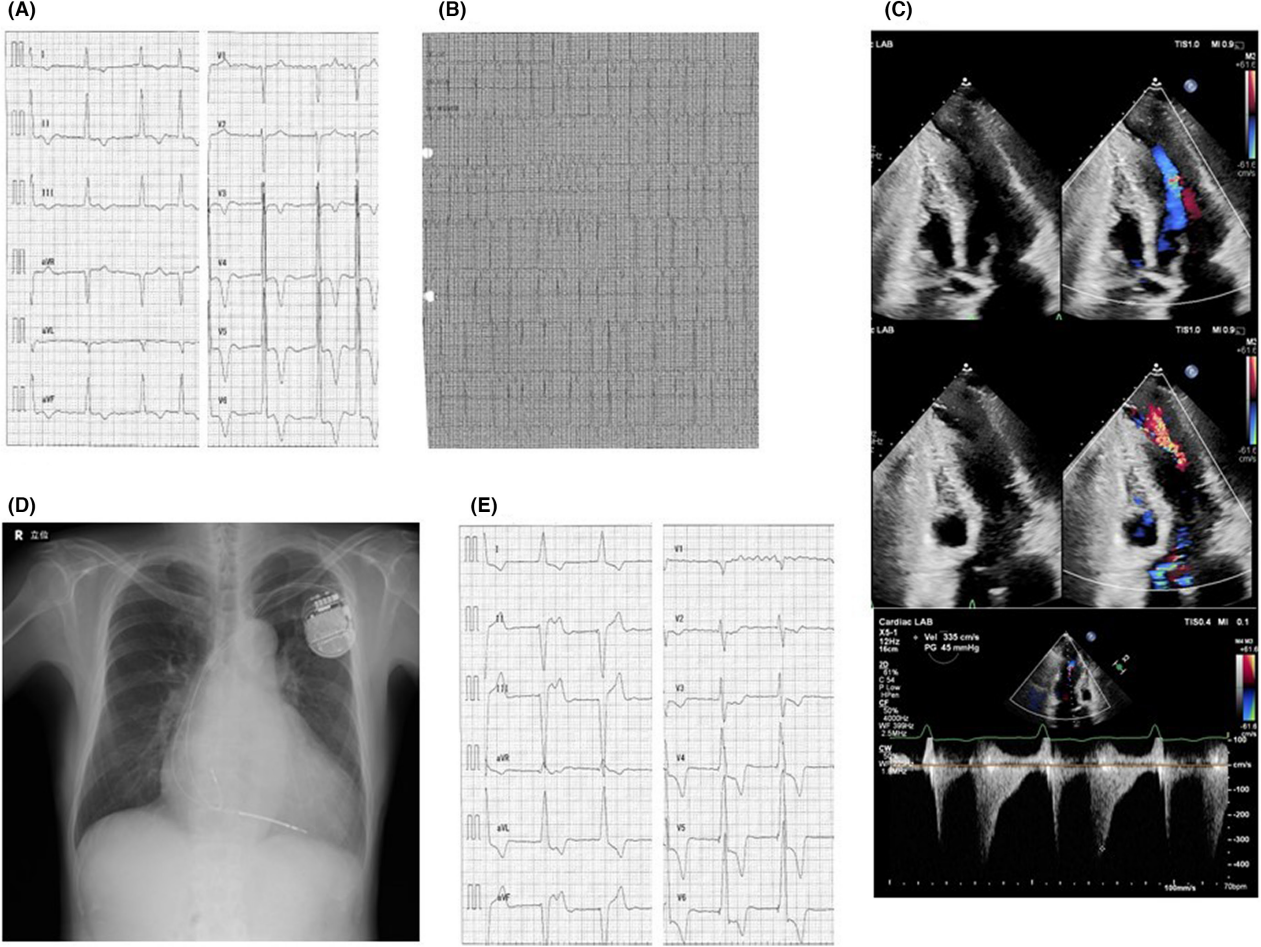
(A) The 12‐lead electrocardiogram (ECG). He had persistent atrial fibrillation. (B) A Holter ECG of nonsustained ventricular tachycardia. (C) Ultrasonic echocardiography (UCG) revealing left mid‐ventricular obstruction with a 24 mm maximum left ventricular wall thickness and a 45 mmHg intraventricular pressure gradient. The apex of the left ventricle was aneurysmal and thinning. (D) Chest x‐ray after ICD implantation. The right ventricular lead was placed in the right ventricular apex. (E) ECG during RV pacing.

All three patients had been using β‐blockers prior to their visit to our hospital. Ventricular leads were placed in the right ventricular apex (Figures 1D, 2C, and 3D). No new antiarrhythmic drugs such as amiodarone were used after ICD implantation. After ICD implantation, right ventricular pacing was initiated (Figures 1E, 2D, and 3E). In addition, they were able to ascend stairs immediately after implantation. Figure 4 shows the serial changes in intraventricular pressure gradient in the left ventricle and New York Heart Association functional classification in each case. In one case, the left intraventricular pressure gradient disappeared immediately after right ventricular pacing, while in the others it disappeared during the chronic phase, more than a year later. Symptoms such as shortness of breath on exertion improved in all patients.

**FIGURE 4:**
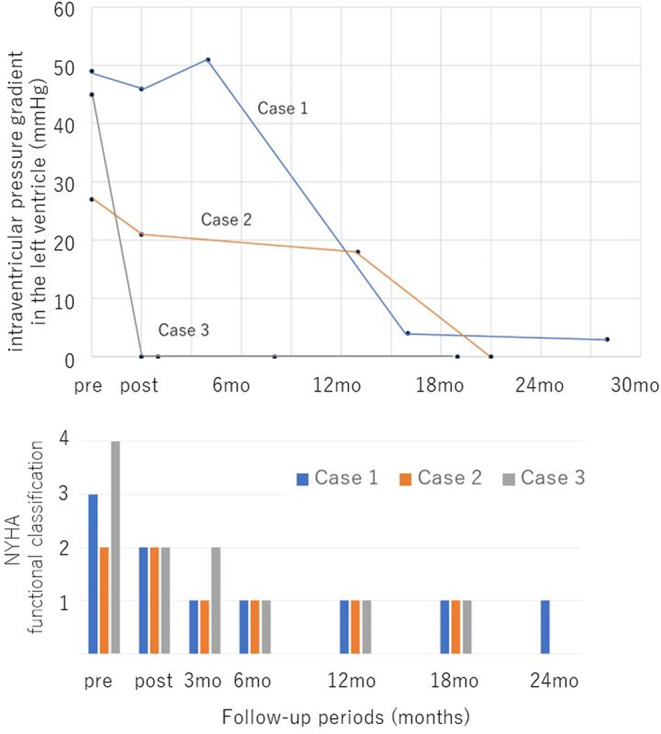
The serial changes in intraventricular pressure gradient in the left ventricle and New York Heart Association (NYHA) functional classification in each case. Both the left intraventricular pressure gradient and symptoms improved after right ventricular pacing.

Right ventricular pacing therapy not only reduced the intraventricular pressure gradient in the left ventricle, but also improved symptoms in our three cases with symptomatic LMVO. High pressure is applied to the left ventricular apex as in our cases as the stage of LMVO progresses, resulting in a left ventricular apex aneurysm. Therefore, pacing therapy can relieve the high pressure in the left ventricular apex and prevent it from progressing to a left ventricular apex aneurysm in patients with LMVO. Moreover, it might prevent ventricular arrhythmia development.

Sakai et al.^1^ reported hypertrophy regression after chronic right ventricular pacing. A similar finding was seen in our first case. The pressure gradient reduction might reduce pressure overload of the LV wall, which change might not only prevent but also improve left ventricular wall thickening.

The JCS/JHRS 2019 Guideline on Non‐Pharmacotherapy of Cardiac Arrhythmias^4^ recommends that permanent pacemaker implantation should be considered for patients with significant LVOT pressure gradient associated with quality of life deterioration. However, the JCS/JHFS 2018 Guideline on the Diagnosis and Treatment of Cardiomyopathies^5^ regarded septal reduction therapy (SRT), such as surgical septal myectomy and percutaneous transluminal septal myocardial ablation, as invasive treatments for symptomatic drug‐resistant HOCM. However, this guideline described that pacing therapy is distinguished from SRT and is described as another invasive treatment. ICD implantation was indicated in our three cases, thus pacing therapy was performed after implantation and was very effective. Therefore, SRT was not necessary in these three cases.

As for the mechanism of this pressure gradient improvement in our cases, similar to LVOT obstruction, RV pacing may cause desynchronization within the left ventricle, especially in the hypertrophied area, which reduces pressure gradient. However, only three cases were examined in this article, and further studies are needed.

In conclusion, right ventricular pacing is effective for symptomatic LMVO with improvement in intraventricular pressure gradient in the left ventricle and their symptoms.

## CONFLICT OF INTEREST STATEMENT

The authors declare no conflicts of interest.
